# Racial/ethnic disparities in the distribution of heatwave frequency and expected economic losses in the US

**DOI:** 10.1038/s41598-024-67760-w

**Published:** 2024-07-24

**Authors:** Jayajit Chakraborty

**Affiliations:** Bren School of Environmental Science and Management, University of California, Santa Barbara, USA

**Keywords:** Environmental social sciences, Natural hazards

## Abstract

Previous research on social disparities in heat exposure has not examined heatwave frequency or economic damage at the local or neighborhood level. Additionally, most US studies have focused on specific cities or regions, and few national-scale studies encompassing both urban and rural areas have been conducted. These gaps are addressed here by analyzing racial/ethnic disparities in the distribution of annual heatwave frequency and expected economic losses from heatwave occurrence in the contiguous US. Census tract-level data on annualized heatwave frequency and expected loss from the FEMA’s National Risk Index are linked to relevant variables from the American Community Survey. Results indicate that all racial/ethnic minority groups except non-Hispanic Black are significantly overrepresented in neighborhoods with greater annual heatwave frequency (top 10% nationally), and all minority groups are overrepresented in neighborhoods with greater total expected annual loss from heatwaves, compared to non-Hispanic Whites. Multivariable models that control for spatial clustering, climate zone, and relevant socio-demographic factors reveal similar racial/ethnic disparities, and suggest significantly greater heatwave frequency and economic losses in neighborhoods with higher percentages of Hispanics and American Indians. These findings represent an important starting point for more detailed investigations on the adverse impacts of heatwaves for US minority populations and formulating appropriate policy interventions.

## Introduction

The intensity, frequency, and duration of extreme heat events has increased because of climate change^1^. Extreme heat has been the greatest weather-related cause of death in the US in recent decades, resulting in an average over 5600 deaths annually^2^. Under the rubric of thermal inequity research^3^, empirical studies have found racial/ethnic minorities, individuals of lower socioeconomic status, and other vulnerable groups to be disproportionately exposed to extreme heat and its adverse effects. Heat-related social inequities in the US have been linked to discriminatory housing, urban development, and planning policies that promote spatial and racial/ethnic segregation, and restrict residential choices of non-White minorities and low-income individuals to hotter neighborhoods with limited greenspace and tree canopy cover^4–7^.

Heat exposure and related impacts have been measured in thermal inequity studies using land surface temperature^5,6,8–10^, surface urban heat island intensity^11,12^, urban heat risk indices^4,13^, heat vulnerability indicators^7,14^, heat-related hospitalization^15^, and mortality^16^. The frequency of local heatwaves and resulting economic damage have not been investigated in previous thermal inequity research. Additionally, most US-based studies have focused on specific cities, states, or regions, and few national-scale studies encompassing both urban and rural areas have been conducted. This study aims to address these gaps and extend thermal inequity research by analyzing the distribution of both annual heatwave frequency and expected economic damage from heatwave occurrence in the contiguous US (lower 48 states and Washington DC). Two research questions are examined: (1) Are racial/ethnic minority groups significantly overrepresented in neighborhoods with the highest heatwave frequency and total expected loss from heatwaves (top 10% nationally), compared to non-Hispanic Whites? (2) How do the racial/ethnic composition of neighborhoods relate to heatwave frequency and expected losses, after accounting for relevant factors known to influence heat exposure and geographic clustering?

Census tract-level data on heatwaves is derived from the US Federal Emergency Management Agency (FEMA)’s National Risk Index (NRI)^17^. Heatwaves are defined in the NRI as a period of abnormally and uncomfortably hot and unusually humid weather for a given area, typically lasting two or more days, with temperatures (as defined by the area’s local weather forecast office) outside the historical averages. Modeled estimates of annualized heatwave frequency and total expected annual loss (combines and quantifies losses for three consequence types: agriculture, building, and human fatalities/injuries) are linked to relevant variables from the American Community Survey (ACS) five-year estimates.

## Results

**Figure 1:**
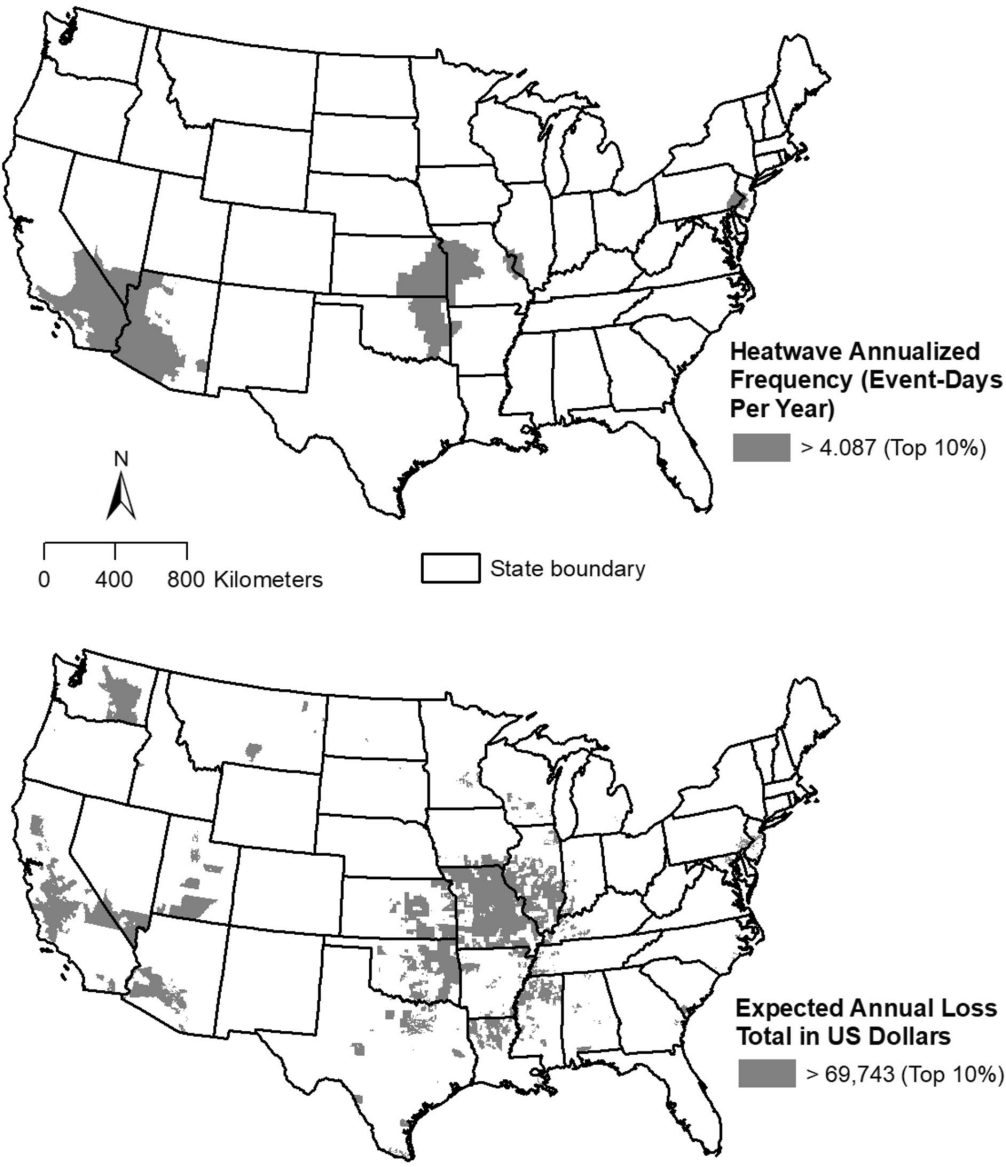
Census tracts in the 90th percentile or above (top 10%) in the contiguous US by: (**a**) National Risk Index (NRI) modeled heatwave annualized frequency; and (**b**) total expected annual loss from heatwave occurrence, 2022.

Figure 1 depicts the distribution of census tracts in the top 10% (at or above the 90th percentile) for annualized heatwave frequency and total expected loss from heatwave occurrence, respectively, in the contiguous US. These tracts are concentrated mainly in southwestern states (e.g., Arizona and California), as well as those in the lower Midwest (e.g., Kansas and Missouri).

**Figure 2:**
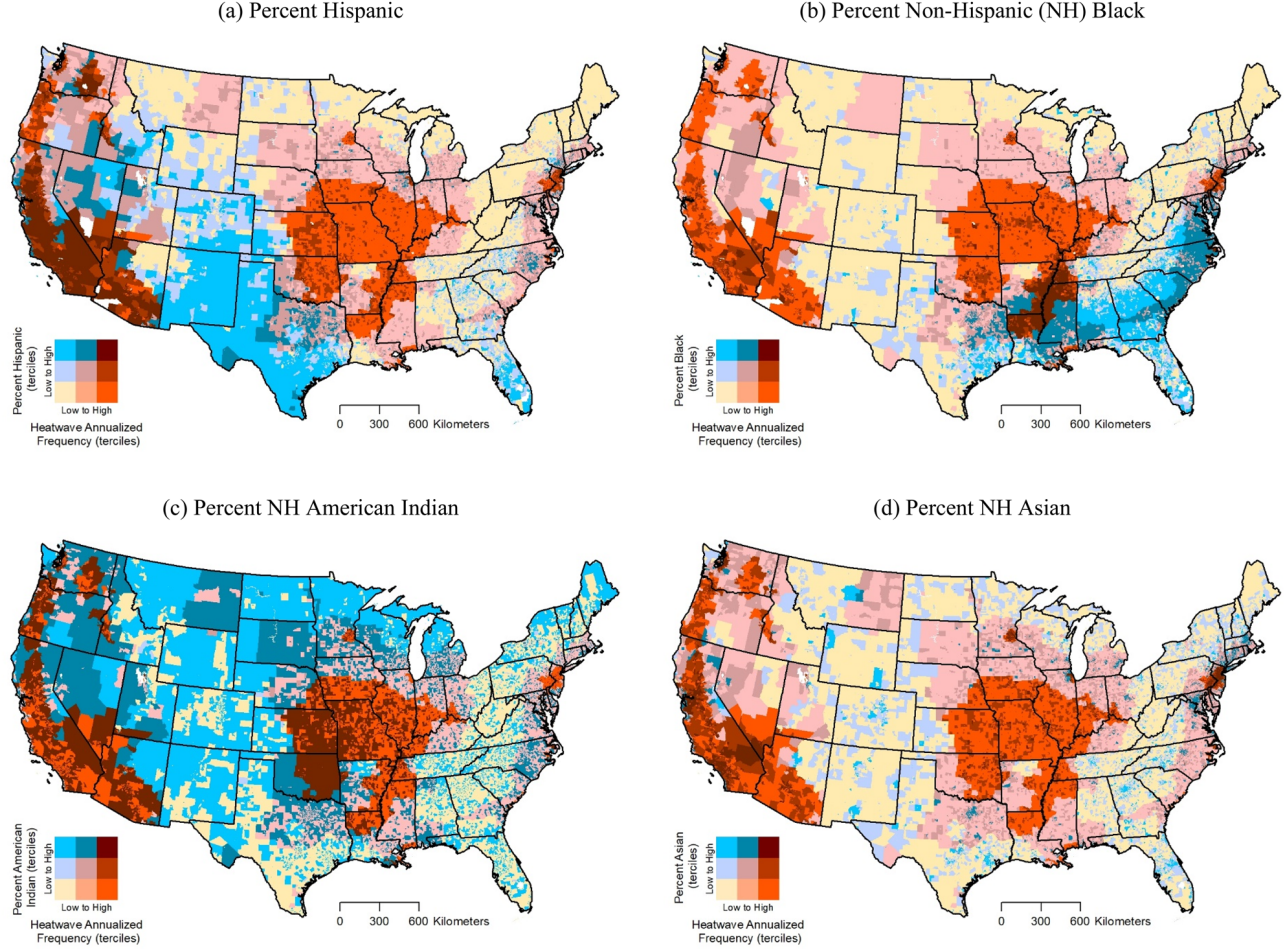
Relationship between National Risk Index (NRI) modeled heatwave annualized frequency and major racial/ethnic minority groups by census tract.

**Figure 3:**
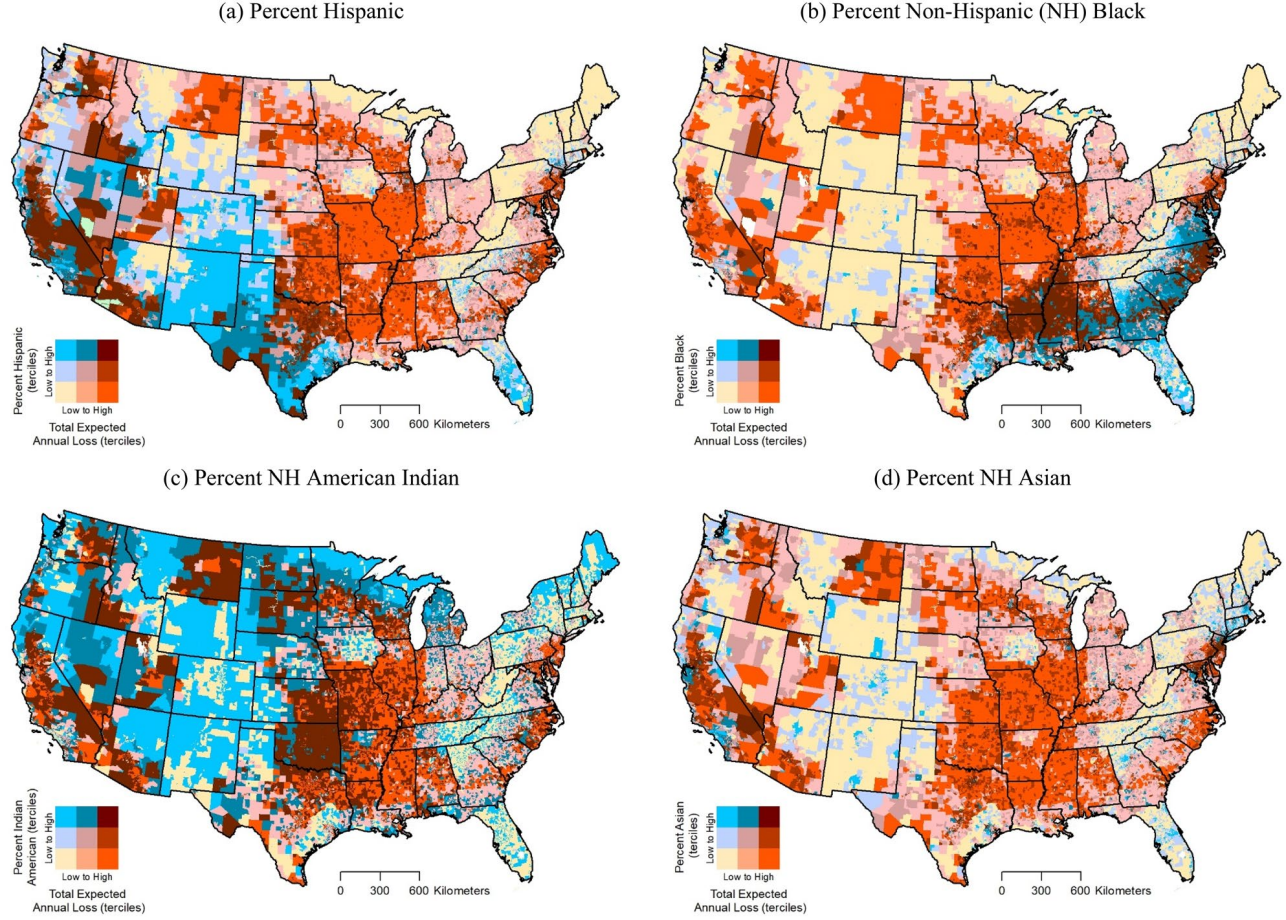
Relationship between NRI modeled total expected annual loss from heatwave occurrence and major racial/ethnic minority groups by census tract.

Figure 2 shows how relationships between annualized heatwave frequency and specific racial/ethnic minority groups vary spatially across the US. These bivariate maps suggest that tracts in the highest tercile (top 33.3%) for both variables are located mainly in the West (e.g., California) for Hispanic percentage and the South (e.g., Arkansas, Louisiana, and Mississippi) for non-Hispanic Black percentage. Tracts in the highest tercile for both heatwave frequency and American Indian percentage indicate a more dispersed pattern, and can be found in the West, Midwest, and South (e.g., California, Nevada, Kansas, and Oklahoma). The spatial distribution of relationships between total expected annual loss from heatwaves and racial/ethnic minority groups are shown in Fig. 3. Tracts in the highest tercile (top 33.3%) for both variables can be found in multiple states of the West for Hispanic percentage and South for non-Hispanic Black percentage. Tracts in highest tercile for both total expected loss and American Indian percentage again suggest a more dispersed pattern and are located in multiple US regions (e.g., West, Midwest, and South).

Statistical results from racial/ethnic comparisons are summarized in Table 1, where each minority group’s percentage (based on their national total) in the top decile for each heat indicator is compared to the corresponding non-Hispanic White percentage. For tracts in the top 10% for heatwave frequency (Figure 1a), risk ratios (RRs) significantly exceed 1.0 (*p* < 0.001) for all minority groups, except non-Hispanic Black. Specifically, Hispanic, American Indian, and Asian persons are 71.12%, 105.4% and 45.4% more likely, respectively, to reside in these top 10% tracts, while Black residents are 5.7% less likely (compared to non-Hispanic Whites). For tracts in the top 10% nationally for total expected annual loss (Figure 1b), RRs for all minority groups significantly exceed 1.0 (*p* < 0.001). Specifically, Hispanic, non-Hispanic Black, American Indian, and Asian persons are 16.5%, 34.9%, 49.1%, and 3.6% more likely, respectively, to reside in these tracts. American Indians indicate the greatest overrepresentation (highest RRs) with respect to non-Hispanic Whites within tracts in the top 10% for both heatwave frequency and total expected loss.

**Table 1:** Distribution of persons by race/ethnicity in tracts in the top 10% for National Risk Index (NRI) modeled heatwave annualized frequency (event-days per year) and total expected annual loss from heatwave occurrence (US dollars).

	Tracts in top 10% of heatwave annualized frequency				Tracts in top 10% of total expected annual loss from heatwave occurrence			
	Percent (%)	Risk ratio	95% CI of risk ratio	Z-test statistic (*p* value)	Percent (%)	Risk ratio	95% CI of risk ratio	Z-test statistic (*p* value)
Hispanic (of any race)	15.06	1.712	[1.711, 1.713]	1406.525 (< 0.001)	12.41	1.165	[1.164, 1.166]	382.200 (< 0.001)
Non-Hispanic (NH) Black	8.29	0.943	[0.942, 0.944]	102.584 (< 0.001)	14.37	1.349	[1.348, 1.350]	683.737 (< 0.001)
NH American Indian	18.07	2.054	[2.048, 2.061]	452.631 (< 0.001)	15.88	1.491	[1.486, 1.496]	233.090 (< 0.001)
NH Asian	12.79	1.454	[1.452, 1.456]	568.975 (< 0.001)	11.04	1.036	[1.035, 1.038]	50.721 (< 0.001)
NH Multi/Other minority Race	12.50	1.421	[1.418, 1.423]	465.136 (< 0.001)	12.08	1.134	[1.132, 1.135]	164.820 (< 0.001)
NH White [reference group]	8.80				10.65			

N = 83,481 census tracts in contiguous US. Risk ratios (RRs) are based on dividing minority group percentages (based on their total population in continental US) by corresponding NH White percentage.

Results from multivariable generalized estimating equation models for predicting annualized heatwave frequency and total expected annual loss, respectively, based on racial/ethnic characteristics and a set of control variables are presented in Table 2. After accounting for spatial clustering of tracts, relevant socio-demographic factors known to be associated with extreme heat exposure in the US^7,10^, and climate zones defined by the U.S. Department of Energy, annualized heatwave frequency indicates a statistically significant and positive relationship (*p* < 0.05) with the percentages of Hispanic and American Indian residents. All racial/ethnic groups reveal significantly positive associations (*p* < 0.05) with total expected annual loss, with the exception of non-Hispanic Asians. The percentages of Hispanics and American Indians represent the only racial/ethnic variables to yield a positive and significant (*p* < 0.01) coefficient in both models.

**Table 2:** Multivariable generalized estimating equations for predicting heatwave annualized frequency and total expected annual loss from heatwave occurrence.

Variables	Heatwave annualized frequency (event-days per year)				Total expected annual loss from heatwave occurrence (US dollars)			
	Beta	Lower 95% CI	Upper 95% CI	*p* value	Beta	Lower 95% CI	Upper 95% CI	*p* value
% Hispanic	0.126	0.032	0.220	0.009	0.041	0.026	0.055	< 0.001
% Non-Hispanic (NH) Black	− 0.005	− 0.064	0.053	0.859	0.074	0.064	0.083	< 0.001
% NH American Indian	0.048	0.017	0.080	0.003	0.052	0.043	0.060	< 0.001
% NH Asian	− 0.065	− 0.144	0.014	0.105	0.009	− 0.001	0.019	0.075
% NH multi/other race	0.035	− 0.012	0.082	0.142	0.050	0.042	0.058	< 0.001
Control variables								
% Low-income population	0.003	− 0.051	0.058	0.901	− 0.055	− 0.068	− 0.043	< 0.001
% Less than high school education	− 0.016	− 0.076	0.043	0.594	− 0.014	− 0.028	− 0.001	0.040
% Unemployed population	0.012	− 0.037	0.061	0.623	0.012	0.003	0.021	0.011
% Limited English speaking	− 0.009	− 0.074	0.056	0.784	− 0.007	− 0.019	0.006	0.279
% With a disability	0.018	− 0.025	0.062	0.414	− 0.002	− 0.014	0.009	0.671
% Age under 5 years	0.049	0.033	0.065	< 0.001	0.094	0.086	0.103	< 0.001
% Age 65 or more years	− 0.034	− 0.066	− 0.002	0.040	− 0.082	− 0.093	− 0.072	< 0.001
Population density (persons/sq. mile)	0.019	− 0.035	0.073	0.495	0.036	0.028	0.044	< 0.001
Metropolitan status (1/0)	0.373	0.223	0.524	< 0.001	0.078	0.054	0.103	< 0.001
Hot dry climate zone	1.995	1.302	2.687	< 0.001	0.530	0.501	0.560	< 0.001
Hot humid climate zone	− 0.616	− 0.915	− 0.316	< 0.001	0.140	0.114	0.166	< 0.001
Marine climate zone	0.563	0.286	0.839	< 0.001	− 1.432	− 1.488	− 1.376	< 0.001
Mixed dry climate zone	0.289	− 0.463	1.042	0.451	0.039	− 0.056	0.133	0.421
Mixed humid climate zone	0.741	0.517	0.964	< 0.001	0.730	0.710	0.750	< 0.001
Intercept	− 0.555	− 0.748	− 0.362	< 0.001	9.796	9.770	9.821	< 0.001

N = 82,747 tracts in contiguous US with at least 500 persons. Both models use an independent correlation matrix and Tweedie (index parameter = 1.5) distribution with logarithmic link function, and adjust for clustering based on the county in which tract is located (3101 clusters). Numbers in the ‘*p* value’ column represent two-tailed *p* values from the Wald Chi-Square test. All continuous independent variables were standardized before model entry. Reference categories include ‘% Non-Hispanic White’ for racial/ethnic groups and ‘Very cold/cold’ climate zones for the U.S. Department of Energy climate zone designation.

Expected annual losses associated with the three consequence types that comprise total expected annual loss were also analyzed separately. Results from multivariable models focusing on expected losses related to agriculture, buildings, and population equivalence (inflation-adjusted dollar value assigned to the sum of human fatalities and injuries), respectively, are summarized in Table 3 (control variables not shown). Expected annual loss for agriculture reveals a significantly positive association (*p* < 0.001) with the percentages of Hispanic and American Indian residents, but a negative association (*p* < 0.001) with other racial/ethnic variables. Expected annual loss related to buildings indicates a significantly negative (*p* < 0.001) or non-significant association (*p* > 0.05) with all racial/ethnic variables. In contrast, expected annual loss to the population shows a significant and positive relationship (*p* < 0.001) with all five racial/ethnic minority groups. Hispanics and American Indians represent the only racial/ethnic minority groups to indicate a significantly positive association with expected annual losses from two consequence types: agriculture and population equivalence.

**Table 3:** Multivariable GEEs for predicting expected annual loss from heatwave occurrence by consequence type.

	Beta	Lower 95% CI	Upper 95% CI	*p* value
Dependent variable: Expected annual loss (USD)	Agriculture			
% Hispanic	0.609	0.599	0.618	< 0.001
% Non-Hispanic (NH) Black	− 1.787	− 1.811	− 1.762	< 0.001
% NH American Indian	0.171	0.165	0.177	< 0.001
% NH Asian	− 0.487	− 0.503	− 0.472	< 0.001
% NH multi/other race	− 0.194	− 0.209	− 0.180	< 0.001
Dependent variable: Expected annual loss (USD)	Buildings			
% Hispanic	− 0.120	− 0.139	− 0.101	< 0.001
% Non-Hispanic (NH) Black	− 0.330	− 0.343	− 0.318	< 0.001
% NH American Indian	− 0.092	− 0.102	− 0.081	< 0.001
% NH Asian	− 0.292	− 0.306	− 0.278	< 0.001
% NH multi/other race	− 0.007	− 0.016	0.003	0.163
Dependent variable: Expected annual loss (USD)	Population equivalence			
% Hispanic	0.050	0.036	0.065	< 0.001
% Non-Hispanic (NH) Black	0.080	0.071	0.089	< 0.001
% NH American Indian	0.045	0.036	0.054	< 0.001
% NH Asian	0.026	0.016	0.036	< 0.001
% NH multi/other race	0.057	0.048	0.065	< 0.001

N = 82,747 tracts in contiguous US with at least 500 persons. All three GEE models use an independent correlation matrix and Tweedie (index parameter = 1.5) distribution with logarithmic link function, and adjust for clustering based on the county in which tract is located (3101 clusters). Numbers in the ‘*p* value’ column represent two-tailed *p* values from the Wald Chi-Square test. Each multivariable model includes the racial/ethnic groups listed above as independent variables and the following control variables: % Low-Income population, % Less than high school education, % Unemployed population, % Limited English speaking, % With a disability, % Age under 5 years, % Age 65 or more years, population density (persons per sq. mile), metropolitan status (1/0), and U.S. Department of Energy climate zone designations (Hot-Dry, Hot-Humid, Marine, Mixed-Dry, and Mixed-Humid). Reference categories include ‘% Non-Hispanic White’ for racial/ethnic group and ‘Very Cold/Cold’ for the U.S. Department of Energy climate zone designation.

## Discussion

This study extends thermal inequity research by conducting a national-scale analysis of racial/ethnic disparities in local heatwave frequency and expected economic loss from heatwave occurrence—indicators that have not been examined previously. The first research question sought to determine whether racial/ethnic minority groups are overrepresented in neighborhoods with the highest heatwave frequency and total expected loss from heatwaves (i.e., tracts in the top 10% nationally), compared to the non-Hispanic White population. Results indicate that all racial/ethnic minority groups except non-Hispanic Black are significantly overrepresented in neighborhoods with the highest heatwave frequency (top 10%), and all minority groups examined are overrepresented in neighborhoods with the highest total expected loss.

The second research question focused on analyzing racial/ethnic disparities in the distribution of annualized heatwave frequency and expected loss in the contiguous US, after controlling for the effects of socioeconomic factors, younger and older age, population density, metropolitan status, climate zone, and spatial clustering by US county. Multivariable models indicated increased heatwave frequency in neighborhoods with higher percentages of Hispanic and American Indian residents. Expected economic loss from heatwaves is found to increase significantly in neighborhoods with higher percentages of Hispanic, Black, American Indian, and other non-Hispanic minorities who are not Asian. Thus, neighborhoods characterized by greater American Indian prevalence are disproportionately impacted by both higher heatwave frequency and economic damage. When total economic loss from heatwave occurrence was disaggregated by consequence type, racial/ethnic disparities were found to persist for damages related to agriculture and population (i.e., dollar-based summation of fatalities and injuries). While expected agricultural loss was significantly greater in neighborhoods with higher Hispanic and American Indian percentages, expected population loss was found to increase significantly in neighborhoods with higher percentages of all five racial/ethnic minority groups.

Findings from this study related to annual heatwave frequency are consistent with previous thermal inequity research that found significant racial/ethnic disparities in the spatial distribution of extreme heat in specific regions and urban areas of the US, based on land surface temperature^8,10^, urban heat island intensity^11,12^, urban heat risk indices^4^, heat-related hospital admissions^15^, and other heat vulnerability indicators^7^. The analysis of expected annual losses, however, provides new and important insights on how specific racial/ethnic minority groups are disproportionally impacted by the consequences of heatwave occurrence. Results of this study indicate that the geography of racial/ethnic disparities in total economic loss is strongly influenced by the spatial distribution of fatalities and injuries suffered by the resident population, and to a lesser extent, the distribution of agricultural losses. Since population-related losses are positively related to the proportions of all racial/ethnic minority groups even after controlling for socioeconomic status and other contextual factors, policies, programs, and strategies that address the negative health impacts of heatwave events in minority communities must be prioritized.

Although previous research has highlighted thermal burdens imposed on Black and Hispanic populations in specific US regions and urban areas, this study indicates that Hispanics and Indian Americans are particularly more likely to reside in areas exposed to significantly greater heatwave frequency, agricultural losses, and population-related losses, at the national scale. From a thermal inequity perspective, these are disturbing results since both these minority groups are characterized by lower socioeconomic status and healthcare access. Vulnerability of Hispanic individuals to heatwave consequences is potentially amplified because Hispanics are disproportionately represented in the outdoor workforce^18^ and more likely to lack health insurance compared to other racial/ethnic groups^19^. Since Native Americans are also known to be socioeconomically disadvantaged^20^ and face significant inequities in both health care and health status^21^, they are likely to have limited coping capacity and higher vulnerability to heatwave exposure and economic losses than other groups. The findings for this study thus emphasize the need for additional research on thermal inequities experienced by Hispanic and Native American communities in the US, as well as policy interventions that provide equitable protection from the adverse impacts of heatwaves.

While this study represents an important first step in analyzing racial/ethnic disparities associated with heatwave occurrence and its local economic impacts, it is important to consider a few limitations. First, using a global multivariable model to analyze statistical associations for this national scale analysis can mask regional or local variations in relationships between the dependent and independent variables. Future research should employ spatial analytic approaches that can be used to explore spatial non-stationarity of model parameters, and identify hotspots where specific minority groups are exposed to elevated levels of heatwave occurrence and economic losses. Second, this analysis did not consider heat mitigation variables (e.g., air conditioning or tree shade) due to limited availability of reliable nationwide data. Third, the results are limited to tract-level associations because FEMA’s NRI data on heatwave exposure and expected losses are unavailable for smaller spatial units. Since heatwave frequency and economic damage are disproportionately distributed with respect to racial/ethnic minority groups at the neighborhood level, more individual and household level analyses that combine quantitative and qualitative data^22,23^ are recommended to examine the adverse consequences of heatwaves exposure and formulate appropriate policy interventions that focus on protecting the health and safety of US minority populations, and American Indian communities, in particular.

## Methods

This study was based on publicly available and aggregated datasets described below, and no humans or living organisms were involved.

### Datasets

Data associated with the two heatwave variables were obtained from the FEMA’s NRI [https://hazards.fema.gov/nri/data-resources#csvDownload], a dataset and tool for identifying communities at risk to 18 natural hazards^17^. Modeled census tract-level values of: (1) annualized heatwave frequency; and (2) total expected annual loss (EAL) from the latest NRI (March 2023); are used as dependent variables. In the NRI, annualized frequency of a natural hazard is defined as the expected frequency or probability of a hazard occurrence per year. The EAL for any natural hazard is calculated on the basis of a multiplicative equation that considers the consequence risk factors of natural hazard exposure, historic loss estimates, and annualized frequency of the hazard.

Annualized heatwave frequency in an area is estimated using the number of recorded heatwave occurrences, in event-days, each year over a 16.9 year period of record (11/12/2005–10/06/2022). Total EAL due to heatwave occurrences during the same period are quantified by summing losses associated with agricultural value, building value, and population equivalence (inflation-adjusted dollar value assigned to sum of human fatalities and injuries). Annual data on heatwave frequency and EAL are not available in the NRI for every year in the record period; only aggregated values for the entire timeframe are provided. The statistical analysis also utilized additional dependent variables representing the EAL for each consequence type: agriculture, buildings, and population equivalence. The FEMA’s *NRI Technical Documentation*^24^ provides more information on data sources and methods for tract-level estimation of annualized heatwave frequency and EAL scores.

Data on independent variables representing socio-demographic factors were derived from the 2021 American Community Survey (ACS) five-year estimates, the most recent ACS at the time this study was conducted [https://data.census.gov/table?d=ACS 5-Year Estimates Detailed Tables]. For race/ethnicity, the analysis included variables for the percentage of individuals identified as Hispanic (of any race) and each of the following non-Hispanic groups: White, Black, American Indian, Asian, and multi/other race. Non-Hispanic Whites were included in the bivariate comparisons but excluded from multivariate models to allow results for minority racial/ethnic groups to be interpreted relative to the non-Hispanic Whites. Socio-demographic factors linked to extreme heat exposure in previous US thermal inequity studies^3,7,10^ were included as control variables in multivariate models. These included the percentages of low-income (income less than or equal to twice the federal poverty level), less than high school education, and unemployed individuals, limited English speaking households (all members 14 years or older have some difficulty with English), civilian non-institutionalized persons with a disability, those under the age of 5, and those aged 65 or more years, population density, and if the tract was located in a metropolitan area (1/0). In addition to socio-demographic factors, each model included dichotomous variables (1/0) representing climate zone designations (Hot-Dry, Hot-Humid, Marine, Mixed-Dry, and Mixed-Humid) developed by the US Department of Energy Building program^25^, with the Very Cold or Cold zones combined to serve as the reference group for comparison with other zones.

### Statistical analysis

The analysis for the first research question examines statistical overrepresentation of racial/ethnic minority groups within tracts in the highest decile (top 10% nationally) for both heatwave variables. Comparisons are based on risk ratios (RRs), calculated by dividing the percentage of each minority group (based on their national total) by the corresponding percentage of non-Hispanic Whites. Two-sample z-tests for proportions are used to determine if any minority group percentage differs significantly from the non-Hispanic White percentage.

For the second research question, generalized estimating equations (GEEs) are utilized for multivariable modeling of tract-level associations between relevant heatwave indicators and the entire set of independent variables. By examining the effects of all explanatory factors simultaneously in the same multivariable model, we can identify the extent to which each racial/ethnic variable exerts independent effects on annual heatwave frequency and economic losses, after the impacts of other relevant socio-demographic factors and climate zones are considered. GEEs extend the generalized linear model to account for clustered data^26^ and relax several assumptions of traditional regression (i.e., normality). Both GEE models used here control for clustering based on the US county in which the tract is located (3101 counties). The Tweedie distribution with logarithmic link function and an independent correlation matrix were chosen for these GEEs, since these model specifications provided the best fit based on the quasi-likelihood under the independence model criterion^27^. The statistical significance of variables were based on two-tailed *p* values from Wald’s Chi-Square test. All continuous independent variables were standardized and standardized coefficients are provided in Tables 2 and 3. Diagnostic tests indicated that inferences from the GEEs are not affected by multicollinearity. Tracts in the contiguous US with low population (< 500) were removed before multivariable modeling to ensure stable proportions for all independent variables.

## Data Availability

The datasets used for this study are available from the corresponding author on reasonable request.

## References

[1] Environmental Protection Agency (EPA). *Climate change indicators: Heat waves*; https://www.epa.gov/climate-indicators/climate-change-indicators-heat-waves (2022).

[2] Weinberger, K. R., Harris, D., Spangler, K. R., Zanobetti, A. & Wellenius, G. A. Estimating the number of excess deaths attributable to heat in 297 United States counties. *Environ. Epidemiol.* **4**(3), e096 (2020). 32613153 10.1097/EE9.0000000000000096 PMC7289128

[3] Mitchell, B. C. & Chakraborty, J. Thermal inequity: The relationship between urban structure and social disparities in an era of climate change. In *The Routledge Handbook of Climate Justice* (ed. Jafry, T.) 283–299 (Routledge, 2019).

[4] Mitchell, B. C. & Chakraborty, J. Exploring the relationship between residential segregation and thermal inequity in the 20 U.S. cities. *Local Environ.* **23**, 796–813 (2018). 10.1080/13549839.2018.1474861

[5] Hoffman, J. S., Shandas, V. & Pendleton, N. The effects of historical housing policies on resident exposure to intra-urban heat: A study of 108 US urban areas. *Climate* **8**, 12. 10.3390/cli8010012 (2020). 10.3390/cli8010012

[6] Wilson, B. Urban heat management and the legacy of redlining. *J. Am. Plan. Assoc.* **86**, 443–457 (2020). 10.1080/01944363.2020.1759127

[7] Manware, M., Dubrow, R., Carrión, D., Ma, Y. & Chen, K. Residential and race/ethnicity disparities in heat vulnerability in the United States. *GeoHealth* **6**, e2022GH000695. 10.1029/2022GH000695 (2022). 36518814 10.1029/2022GH000695 PMC9744626

[8] Dialesandro, J., Brazil, N., Wheeler, S. & Abunnasr, Y. Dimensions of thermal inequity: Neighborhood social demographics and urban heat in the southwestern U.S. *Int. J. Environ. Res. Public Health* **18**, 941. 10.3390/ijerph18030941 (2021). 33499028 10.3390/ijerph18030941 PMC7908488

[9] Mashhoodi, B. Temperature rise amplifies environmental inequities? Europe’s north-south divide. *Appl. Spatial Anal.* **17**, 599–617 (2024). 10.1007/s12061-023-09555-6

[10] Renteria, R., Grineski, S., Collins, T., Flores, A. & Trego, S. Social disparities in neighborhood heat in the Northeast United States. *Environ. Res.* **203**, 111805 (2022). 34339695 10.1016/j.envres.2021.111805

[11] Hsu, A. G., Sheriff, T., Chakraborty, D. & Manya, D. Disproportionate exposure to urban heat island intensity across major US cities. *Nat. Commun.* **12**, 2721 (2021). 34035248 10.1038/s41467-021-22799-5 PMC8149665

[12] Johnson, D. P. Population-based disparities in U.S. urban heat exposure from 2003 to 2018. *Int. J. Environ. Res. Public Health* **19**, 12314. 10.3390/ijerph191912314 (2021). 36231614 10.3390/ijerph191912314 PMC9566334

[13] Mitchell, B. C., Chakraborty, J. & Basu, P. Social inequities in urban heat and greenspace: Analyzing climate justice in Delhi, India. *Int. J. Environ. Res. Public Health* **18**, 4800. 10.3390/ijerph18094800 (2021). 33946259 10.3390/ijerph18094800 PMC8124940

[14] Wolf, T. & McGregor, G. The development of a heat wave vulnerability index for London, United Kingdom. *Weather Clim. Extremes* **1**, 59–68 (2013). 10.1016/j.wace.2013.07.004

[15] Guo, C. *et al.* Impact of heat on emergency hospital admission in Texas: Geographic and racial/ethnic disparities. *J. Expo Sci. Environ. Epidemiol.* 10.1038/s41370-023-00590-6 (2023). 37558698 10.1038/s41370-023-00590-6

[16] Monteiro Dos Santos, D. *et al.* Twenty-first-century demographic and social inequalities of heat-related deaths in Brazilian urban areas. *PLoS ONE* **19**, e0295766 (2024). 38265975 10.1371/journal.pone.0295766 PMC10807764

[17] US Federal Emergency Management Agency (FEMA). *The National Risk Index*; https://hazards.fema.gov/nri/ (2023).

[18] Moms Clean Air Force. *Extreme Heat and Latino Communities Factsheet*; https://www.momscleanairforce.org/resources/extreme-heat-and-latino-communities/ (2022).

[19] Keisler-Starkey, K. & Bunch, L. N. *Health Insurance Coverage in the United States: 2019*. https://www.census.gov.

[20] Hathaway, E. D. American Indian and Alaska Native people: Social vulnerability and COVID-19. *J. Rural Health.* **37**, 256–259 (2021). 32744339 10.1111/jrh.12505 PMC7436677

[21] Smith, M. Native Americans: A crisis in health equity. *Hum. Rights Mag.* **43**, 68 (2017).

[22] Bryne, J. *et al.* Could urban greening mitigate suburban thermal inequity?: The role of residents’ dispositions and household practices. *Environ. Res. Lett.* **10**, 095014 (2016). 10.1088/1748-9326/11/9/095014

[23] Lanza, K. *et al.* Heat vulnerability of Latino and Black residents in a low-income community and their recommended adaptation strategies: A qualitative study. *Urban Clim.* **51**, 101656. 10.1016/j.uclim.2023.101656 (2023). 10.1016/j.uclim.2023.101656

[24] Zuzak, C. *et al.**National Risk Index Technical Documentation* (Federal Emergency Management Agency, 2023).

[25] U.S. Energy Information Administration, *Climate Zones—U.S. Department of Energy (DOE) Building America Program*. https://atlas.eia.gov/datasets/eia::climate-zones-doe-building-america-program/about (2023).

[26] Liang, K. Y. & Zeger, S. L. Longitudinal data analysis using generalized linear models. *Biometrika* **73**, 13–22 (1986). 10.1093/biomet/73.1.13

[27] Garson, G. *Generalized Linear Models and Generalized Estimating Equations* (Statistical Associates Publishing, 2021).

